# Digital Transformation in Patient Organizations: Interview and Focus Group Study

**DOI:** 10.2196/62750

**Published:** 2025-02-13

**Authors:** Simon Wallraf, Sara Köthemann, Claudia Wiesemann, Sabine Wöhlke, Marie-Luise Dierks, Marion Andrea Schmidt, Henk Jasper van Gils-Schmidt, Jonas Lander

**Affiliations:** 1 Institute for Epidemiology, Social Medicine and Health Systems Research Hannover Medical School Hanover Germany; 2 Department of Medical Ethics and History of Medicine University Medical Center Göttingen Göttingen Germany; 3 Department of Health Sciences Hamburg University of Applied Sciences Hamburg Germany

**Keywords:** patient organization, patient support, digitalization, digital transformation, health research

## Abstract

**Background:**

Patient organizations (POs) are an integral part of the health care landscape, serving as advocates and support systems for patients and their families. As the digitalization of health care accelerates, POs are challenged to adapt their diverse roles to digital formats. However, the extent and form of POs’ digital adaptation and the challenges POs encounter in their digital transformation remain unexplored.

**Objective:**

This study aims to investigate the digital transformation processes within POs. We examined the types of digital activities and processes implemented, people involved in respective tasks, challenges encountered, and attitudes toward the digitalization of POs.

**Methods:**

The study was carried out by the multicenter interdisciplinary research network Pandora. We adopted a qualitative exploratory approach by conducting 37 semistructured interviews and 2 focus groups with representatives and members of POs in Germany. Results were obtained using a deductive-inductive approach based on a qualitative content analysis. Methods and results were reported in accordance with the COREQ (Consolidated Criteria for Reporting Qualitative Research) checklist.

**Results:**

POs primarily apply basic digital tools to engage in communication, health education, and information dissemination. Some also develop specific mobile apps and collect health data through patient registries. Volunteers cover a considerable part of the workload. Sometimes, POs collaborate with external partners, such as health professionals or other nonprofit organizations. Furthermore, many (13/46, 28%) interviewees referred to the importance of involving members in digitalization efforts to better meet their needs. However, they described the actual practices used to involve members in, for example, developing digital services as limited, passive, or implicit. When evaluating digital transformation processes, representatives and members of POs expressed generally positive attitudes and acknowledged their potential to improve the accessibility of support services, management efficiency, and outreach. Still, resource constraints; the complexity of digital initiatives; and accessibility issues for certain demographic groups, especially older persons, were frequently mentioned as challenges. Several (15/46, 33%) interviewees highlighted POs’ increasing responsibility to support their members’ digital competencies and digital health literacy.

**Conclusions:**

POs are actively involved in the digital transformation of health services. To navigate challenges and further shape and sustain digital activities and processes, POs may benefit from governance frameworks, that is, a clear plan outlining with whom, how, and with what objectives digital projects are being realized. Support from public, scientific, and policy institutions to enhance the process through training, mentorship, and fostering collaborative networks seems warranted.

## Introduction

### Background

The digital transformation of health care is an ongoing process in which health care–related services, such as types of care, diagnostic methods, and health information, are being digitalized [1-3]. The degree of digitalization varies considerably between different health care organizations and health sectors, and this gap has widened during the COVID-19 pandemic. This is due to, inter alia, the available resources and organizational structures, the degree of digital literacy of those responsible for implementing digitalization, and attitudes toward digital services [4,5]. Some institutional actors within the health care system, such as health insurance companies, ask individuals to contribute to the digital transformation and develop their own digital literacy.

It has been argued that the development of individual skills and attitudes toward digital transformation requires health care institutions to foster comprehensive governance frameworks that specify necessary goals, needs, and methods [3]. Furthermore, an individual’s health is typically not managed by a single health care professional but rather involves multiple entities. To make this possible, these institutions need to provide the necessary resources and define the appropriate levels of contribution and literacy [3]. Overall, digital transformation requires more collective efforts across the health care sector to counter the uneven degrees of digital transformation [5].

Alongside primary care services, patient organizations (POs) are now recognized as key health advocates, providing vital support to patients, people with chronic conditions, and their families. While varying in size and scope, all POs aim to empower individuals to better manage their condition, for example, by providing health information and self-management resources [6,7]. Previous research shows that POs represent patient voices in health care and health care policy making by developing new forms of collaboration [8-10] and research facilitators and partners [11,12]. In fact, for countries such as the United Kingdom, the Netherlands, Sweden, and Germany, POs are now one of the central contributors to health care research, for example, when their members contribute to the planning and implementation of study projects. The main domain in which this takes place is rare disease research [13,14].

Today, POs face the need to join in the digital transformation of society and the medical field specifically [15]. Furthermore, POs face the challenges associated with shifting to digitalized work processes, for example, the search for appropriate resources. PO-specific challenges, such as the proportion of susceptible and older people who seek help from POs [16-18], add to these demands.

### Objectives

To assist POs in shaping their own digital transformation, a better understanding of current practices, opportunities, and potential barriers is needed, which is, to the best of our understanding, currently largely lacking [12]. Hence, this study aimed to explore the current state of digital transformation in German POs. We aimed to do so by answering four specific research questions (RQs):

1. Which digital projects and services do POs currently implement? (RQ 1)
2. Who are the individuals and institutional actors involved in the process of planning and implementing digital projects and activities? (RQ 2)
3. To what extent and how are PO members involved in digitalization efforts? (RQ 3)
4. Which experiences with and attitudes vis-à-vis digitalization in POs do representatives and members express? (RQ 4)

## Methods

### Study Design and Context

This study was conducted as part of the multicenter research network Pandora [19]. Pandora investigates how POs contribute to the digital health transformation, shape their own digital practices, and address the challenges they face therein [20]. For this study, the 3 study sites pursued distinct, although related, objectives within the same overarching RQs defined by the aim of Pandora. Digitalization practices in POs were explored via semistructured interviews and focus groups. An exploratory design was chosen, as these practices have rarely been investigated so far. We used the COREQ (Consolidated Criteria for Reporting Qualitative Research) checklist to report our methodology (Multimedia Appendix 1) [21].

### Theoretical Foundation

#### Overview

To determine the scope and contents of this study, we narratively summarized current insights related to the roles and tasks of POs nationally and internationally. We used this understanding for our own investigation of the digital transformation within these organizations, that is, to derive broad content-wise foci. We chose this strategy as there has been no qualitative study on the digitalization practices in PO. The available evidence on the *ways of working* of PO centers on 3 main pillars.

#### Tasks and Governance Processes

POs’ routine daily tasks and responsibilities have been described as centering on 4 main aspects: policy development and advocacy (eg, POs represent patient interests vis-à-vis political decision makers), empowerment and education (eg, POs develop and deliver health information on all kinds of chronic and acute health conditions), peer support (eg, POs conduct self-management courses to foster individuals’ coping with chronic diseases), and research (eg, POs collect and provide individual health data from their members for use by clinical researchers) [15]. More generally, previous studies have concluded that, with the evolving existence, role, and scope of work domains of POs, they increasingly prioritize professionalization, such as creating professional management and organizational structures [8].

#### Actor and Member Involvement

Given the breadth of tasks and responsibilities of POs, their efforts to professionalize services and structures and the continuous need for sufficient personnel, technical, administrative, and financial resources, they need to engage in collaboration with other stakeholders [10,22]. Herein, POs not only engage with institutions and organizations but also seek to promote exchange with and involvement of their direct members. To date, this has been most evident in relation to research: POs not only act as intermediaries between research and patients or target groups but also, for example, train their members without a scientific educational background, to become active research partners [12,23].

#### Principles and Values Underlying the Digital Work of POs

So far, the roles and activities attributed to POs, such as competing with other health care stakeholders for resources, using support from industry to develop and maintain their own services, and advocating for patients’ interests, have been described in terms of being directly or indirectly related to ethical aspects [18,24]. For example, POs need to be transparent regarding their partners; they need to balance individual protection and autonomy with collective responsibilities, such as in research advances; and they need to handle rapidly increasing amounts of patient data. However, the distinct challenges and ways in which POs go about their digital transformation have not been studied.

### Target Group

To explore the digital transformation in POs, we invited representatives and members of POs in Germany to participate in this study. Except for the recruitment, no previous relationships with the target group existed. We defined representatives as those persons who work full time, part time, or voluntarily for a PO and are involved in leadership roles. Being affected by a chronic illness or disability was not a requirement to be recognized as a PO representative. PO members are individuals affected by the disease or disability that the PO they are registered with advocates for. It was inconsequential whether they were involved in the PO’s work as long as they were not in a leadership role. We did not include professionals, such as medical staff or clinical researchers. Regarding the sampling procedure, we aimed to include PO representatives and PO members in equal shares. Furthermore, we aimed to approach numerous POs (refer to the Recruitment Strategy section) to increase diversity in terms of geographic location, size, and the specific health conditions or issues they represent. Each study site recruited participants until these criteria were sufficiently met, and no new topics were mentioned by the interviewees.

### Recruitment Strategy

We first conducted a manual web-based search and a comprehensive review of German POs’ umbrella organizations and their member organizations. These findings were then merged into a single list of German POs. As part of this process, we screened the POs’ websites for clear indications of involvement in digital activities beyond merely having a website. POs that did not meet our criteria were excluded, as we focused on organizations where digital transformation processes were already underway, allowing us to explore ongoing practices and experiences in more detail. The final list contained 96 organizations, which were included in a purposive recruitment process at each study center. This included sending invitations by email, making follow-up calls if there was no response to the initial approach, forwarding the invitation to PO members via the POs’ channels of communication, and approaching PO members directly via the Pandora advisory board.

### Data Collection

Qualitative data collection was conducted from October 2022 to April 2023 mainly through individual telephone interviews and complemented by 2 web-based focus groups with PO representatives, the latter to increase practicability for the interviewees (S Wallraf, SK, and HJVGS). All junior and senior research fellows had a background in public health or medical ethics. To develop the semistructured interview guideline, we drafted headlines, ie, main topics according to the literature-based conceptualization of POs outlined earlier in the first step. Then, we added concrete questions for each topic and added new aspects where necessary, that is, to establish the focus on digitalization. While the interview guidelines varied slightly per study site, each included three common sections: (1) general aspects related to the level and relevance of digitalization in the respective PO (eg, digital governance, digital activities, and involved actors); (2) perceived opportunities and challenges of digital technologies (ie, appraisal of principles and values); and (3) the digital transformation of the German health care system (eg, digital governance and involved actors; Multimedia Appendix 2).

During the interviews, we focused on understanding how and in which areas such processes take place. This approach allowed us to capture a range of digitalization practices and to identify patterns of digital activity, ensuring a comprehensive understanding of digitalization. Specific technologies were discussed when mentioned by the interviewees. As the third step in the development of the interview guideline, we gathered feedback on the contents and wording of the draft from the Pandora patient advisory board (n=4). Fourth, we pretested the preliminary interview guidelines with representatives of the target group (study center 1: n=2, 50%; study center 2: n=1, 25%; and study center 3: n=1, 25%). Adjustments were made to the number of questions asked, the interview length, the wording of questions to further adapt them to the target group, and the sequencing of topics. At the beginning of the interviews, for which only the researcher and the participants were present, we provided general information on the researchers’ role in the study without mentioning individual characteristics. We did not conduct repeat interviews and used field notes to structure them.

### Data Analysis

Each audio file was transcribed verbatim to ensure the accuracy of the statements, pseudonymized, and then exported for analysis in MAXQDA 22 (VERBI Software GmbH). To generate our findings, we conducted a qualitative content analysis based on the steps described by Kuckartz [25].

First, we deductively derived the initial main categories for analysis based on our interview guidelines and agreed on these categories among the coauthors. Second, we coded approximately 10% (4/39) of the manuscripts to test the suitability of the main categories. Third, we compared the results of this test phase and further developed and refined the coding system until the main authors agreed (S Wallraf, SK, HJVGS, and JL); refer to the Results section for an overview of the main categories and subcategories. Fourth, we analyzed all remaining manuscripts with the final version of the codebook (S Wallraf, SK, and HJVGS). For this analysis, we used consensual coding; that is, the main person coding discussed any uncertainties with one of the coauthors based on the definitions established for each category in the codebook.

Fifth, once the process of applying the main codes to all manuscripts was complete, we inductively derived subcategories within each main category by screening each code within the main categories to further concretize the content.

Sixth, we added frequencies to each subcategory based on the individual mentions of each aspect to gain an overview of the weight of each aspect mentioned by our participants.

After completing the first full version of the analysis, we presented summaries of the preliminary findings for each main category to the senior Pandora project staff not involved in the data analysis for feedback and interpretation of the findings. Furthermore, the coded segments in the transcripts were peer checked for correct interpretation by those authors who performed the analysis (HJVGS, JL, SK, and S Wallraf) when refining the coding system and during the actual analysis.

### Ethical Considerations

Our study adheres to the Declaration of Helsinki, and ethics approval was obtained from the ethics committees of each study center (project 1: 11_6_22_University Medical Center Göttingen and 2022_20_University of Applied Sciences Hamburg; project 2: 10395_BO_K_2022; and project 3: 11_6_22_University Medical Center Göttingen). All participants consented to their participation before data collection and were given the option to opt out. Participants received no financial compensation. All data were pseudonymized. We did not collect patient data or information.

## Results

### Participant Characteristics

We interviewed 46 participants (n=26, 57% female individuals and n=20, 43% male individuals) who were either a member (n=26, 57%) or a representative, that is, staff (n=20, 43%) of a PO (Table 1).

The mean duration of interviews was 71 (SD 13.7) minutes, and focus groups lasted for 124 minutes. Most (17/46, 37%) interviewees were aged 45 to 59 years, followed by 15 (33%) participants aged ≥60 years. Interviewees often reported having completed secondary (7/46, 15%) or tertiary (32/46, 70%) education, equivalent to a high school or university degree, respectively. The POs, most (18/19, 95%) of them acting at a national level, involved were diverse in size, with 21% (4/19) acting as umbrella organizations (Table 2).

The main themes that were addressed included digital activities (RQ 1), actors involved in POs’ digital transformation (RQ 2), participatory approaches to digital transformation (RQ 3), and current attitudes toward POs’ digital efforts (RQ 4). Overall, POs engaged in a range of digital activities with distinct aims; relied on and engaged in collaboration with volunteers and other institutions to cover the workload; and faced considerable technical, conceptual, and motivational hurdles along the way.

**Table 1:** Characteristics of participants (N=46).

Characteristic		Participants, n (%)
**Age group (y)**		
	18-29	4 (9)
	30-44	10 (22)
	45-59	17 (37)
	≥60	15 (33)
**Sex**		
	Male	20 (43)
	Female	26 (57)
**Educational level**		
	Primary education	1 (2)
	Secondary education	7 (15)
	Vocational training	6 (13)
	Tertiary education	32 (70)
**Status in patient organization**		
	Member	26 (57)
	Staff or representative	20 (43)

**Table 2:** Characteristics of patient organizations (n=19).

Characteristic		Patient organizations, n (%)
**Size (members), n**		
	<100	1 (5)
	100-500	3 (16)
	501-1000	4 (21)
	1001-10,000	3 (16)
	10,001-100,000	3 (16)
	>100,000	1 (5)
	N/A^a^ (umbrella organization)	4 (21)
**Geographic scope**		
	Regional	1 (5)
	National	18 (95)
**Head office location (federal states)**		
	Baden-Württemberg	1 (5)
	Bavaria	1 (5)
	Berlin	5 (26)
	Hesse	2 (11)
	Lower Saxony	1 (5)
	North Rhine-Westphalia	7 (37)
	Rhineland-Palatinate	2 (11)

^a^N/A: not available.

### RQ 1: Digital Activities of POs

The analysis of our interviews and focus groups resulted in 4 primary areas where POs engaged in digital activities: communication, administration, health education, and health research.

#### Subtheme 1.1: Digital Communication

POs mostly used basic communication tools (38/46, 83%), including email, websites, videoconferencing, instant messaging services, and social media, to internally communicate with their members, facilitate communication among members, and externally engage with the wider public (refer to subtheme 1.1 in Table 3).

**Table 3:** Main themes and subthemes mentioned by research participants.

Main theme and subtheme		Example quotes
**Digital activities of POs^a^**		
	1.1. Digital communication	“Internally,... we started with Zoom and now use Teams for communication. We also use WhatsApp for quick coordination and brief information flows, for example with our self-help group leaders or within the board.... Last year, we started using Office 365 with the goal of working centrally through Teams in the future and using Teams groups, so that, for instance, our treasurer has everything she needs available via Teams. The board can also do its work, and when we collaborate with our partners, we can organize joint projects there.”
	1.2. Digital administration	“We now have a membership management system, which is also a program where you can fill in all the member data, including account data and so on. We can now also see online who is affected.”
	1.3. Digital health education	“I know from the PO that they had many offers, especially during the pandemic, where doctors or therapists gave a short lecture on some topic, and where you had the opportunity to submit questions in advance, which were then discussed via the computer.”
	1.4. Digital health research	“I think we are opening up many things with the register that can go more in the direction of digitalization of care.... So we actually developed this as a hybrid of outpatient clinic, software and register software. This means that the doctor can see everything you enter directly. And also graphically... it looks a bit as if it had at least once swum past Apple.”
**PO’s digital (collaboration) actors**		
	2.1. POs as independent actors	“This means that I drive forward all digitalization projects within the organization, for example the connection of a new CM system, which was a very large project, or the relaunch of a new website.... I work full-time in our organization and collaborate with many volunteers in the digitalization department.”
	2.2. Support from volunteers and external partners	“Our homepage is now maintained voluntarily by a member. Whether that's good or bad, I’ll leave that for now. Our Instagram is also managed by a member. Neither of them are on the board. The fact that [it] exists at all was also [their own initiative].”
	2.3. Public and private financial assistance	“The pharmaceutical company even approached us and offered us this sponsoring membership, and for us it is simply a blessing that we can cover our fixed costs... with it.”
**Involvement of PO members**		
	3.1. Motivations to establish and sustain involvement	“It is actually the case that digitalization processes should actually be driven from the bottom up.”
	3.2. Types and intensity of involvement	“... It’s not always easy, there’s a lot of tokenism at both national and international level that you involve patients because there may be funding criteria that require it or because it looks good. And that they are not actually taken seriously.... Of course, we are also involved from time to time when it comes to consultation or consultative processes....”
	3.3. Requirements	“So what can ultimately diminish the motivation to get involved is if you keep making suggestions that are ultimately not taken into account. I mean, there can certainly be good reasons for this, but if, yes, if you have the feeling that nothing is being taken on board, then I think that would probably have a negative effect.”
**Attitudes toward digitalization**		
	4.1. Accelerating management, outreach, and communication	“Young families have also joined us in the meantime.... Of course, that is also a great advantage of digitalization. You can simply put information that is very important online immediately.”
	4.2. Simplifying access to PO support services	“I thought that was great because it’s also a good opportunity for people who don’t have the opportunity to travel that far to go somewhere to take part. People who were previously left out somewhere.”
	4.3. Supporting health research	“Yes, the main reason is because I find it useful for myself and for my treatment, but of course I would like to contribute to making this kind of research possible.”
	4.4. Ineffective and resource-intensive activities	“[If you introduce digital applications] in healthcare, then... they have to generate added value or have a higher benefit than what has been realized in the previous analog processes.... And this has been almost non-existent up to now, apart from perhaps the possibility of booking an appointment with a doctor from your computer at home....”
	4.5. Challenging personal interaction and access	“And of course there’s always the point that we exclude some people who don’t have access to the app. Because they simply can’t cope with it or are somehow very skeptical about digital. Or because they don't have the means. Yes, those are all things like that.”
	4.6. Requiring additional efforts	“Financing is a big issue. So where do I get the financing, who takes care of it and so on, I'm busy with all my hands keeping my organization running, I can’t write to any foundations, so I say there is money but you have to... know where to get it and... we don’t have the manpower at the moment.”
	4.7. Encouraging ambivalence	“Well, I think it’s a development that probably can’t be stopped, because it’s happening in many areas now. So as I said, it depends. Sometimes it’s helpful. Sometimes, I think, you can do without it. And my big concern is data security.”

^a^PO: patient organization.

The aforementioned tools are used by almost all POs, regardless of their size and other digital activities, although not every PO uses all of them combined. In addition, some POs have developed or are developing their own apps or platforms to further support these communication activities. Social media platforms, such as Facebook and Instagram (Meta Platforms, Inc), as well as YouTube (Google LLC) allow POs, for example, to reach a younger and wider audience and disseminate information about the various health care or disease topics they address. While Facebook and Instagram appeared to be relatively established ways of external communication, our interviewees mentioned TikTok (ByteDance) almost never (1/46, 2%), despite the platform’s growing reach. PO members used social media platforms to communicate among themselves. However, 1 (2%) interviewee indicated that their organization does not allow WhatsApp (Meta Platforms, Inc) as a formal channel of communication due to considerations regarding data security. Rather, it is used by members on their own initiative and responsibility. Furthermore, POs have established digital or hybrid meetings to supplement or even replace in-person meetings, overcoming contact restrictions as a consequence of the COVID-19 pandemic, accommodating the disabilities of PO members, and appealing to a wider audience from distinct, that is, more distant places (13/46, 28%).

#### Subtheme 1.2: Digital Administration

Some (9/46, 20%) POs used digital technologies to adapt their administrative and management work (subtheme 1.2 in Table 3). This included managing membership databases digitally, handling bookkeeping via digital systems, generating digital annual financial reports, providing members with digital versions of annual reports, and using collaborative project management software to organize and coordinate their work. Our interview data did not indicate a specific relationship between the use of digital technologies for administrative purposes and the size of the PO. In that sense, digital administration efforts, such as digital membership management, were also found in smaller POs, even if only in a few cases.

#### Subtheme 1.3: Digital Health Education

The increasingly digitalized world entails a new responsibility of POs as digital health educators (22/46, 48%; subtheme 1.3 in Table 3). As repeatedly stated by our interviewees, many PO members had difficulties in effectively searching for and applying health information or using available digital tools to their advantage. Thus, POs followed at least 4 distinct ways to educate their members in the use of digital technologies as part of their goal to support disease management, improve their digital health literacy, and empower them to take part in the digitalized world. First, POs provided digital health information, primarily via their website (11/46, 24%) but also via an app (4/46, 9%), podcast (2/46, 4%), or in an audiovisual format (2/46, 4%), for instance, via YouTube. Second, they organized digital meetings, events, and conferences to inform members about and engage them in the discussions of recent health care topics, particularly those specific to the disease that a respective PO dealt with (7/46, 15%). Third, POs sometimes offered digital training programs and support groups for their members to help them cope with a disease, for instance, through physical exercises and self-management (5/46, 11%). Fourth, they provided education and training on the use of various digital tools as such to enable them to benefit from such services (8/46, 17%). The overall variety of educational services offered to members tended to vary with the size of the PO, with larger POs generally offering a greater range of services.

#### Subtheme 1.4: Digital Health Research

Besides providing digital communication and health education as well as digitalizing their administrative work, some POs have started to develop digital technologies, such as mobile health apps (11/46, 24%) and data registries (12/46, 26%; subtheme 1.4 in Table 3). POs did so either on their own or in cooperation with other stakeholders, such as public research institutes and private companies. Through data registries, POs collected health data from their members to facilitate medical and health care services research. Here, they either forwarded such individual health data to research institutions for further investigation or established mutual research cooperations to work together on the data. Remarkably, some of them also applied these registries to conduct their own research projects, that is, without including an external research partner. The management of a data registry appeared to be related to whether the POs had a specific interest in engaging in research rather than their size and resources.

### RQ 2: Actors Involved in Planning and Implementing POs’ Digital Activities

#### Subtheme 2.1: POs as Independent Actors

When sufficient personnel and financial resources were available, POs advanced their digital transformation independently by permanently employed staff, that is, individuals with either professional, technical, or media expertise (9/46, 20%). These individuals employed by POs were primarily responsible for planning and implementing activities such as digital projects, services, and strategies (subtheme 2.1 in Table 3). Furthermore, it happened that individual organizations relied on regional or national associations of POs (ie, umbrella organizations) that developed technologies for them (9/46, 20%). For example, the German Rheumatism Association developed an app that each partner of that umbrella organization on the regional level could make use of.

#### Subtheme 2.2: Support From Volunteers and External Partners

Owing to a lack of expertise within the POs to develop digital services themselves, interviewees frequently mentioned that they established collaborations with volunteers among their members (13/46, 28%) and external partners (10/46, 22%). Within their own ranks, POs relied on members who, based on their professional background or private interest, had expertise in digital service development and delivery (subtheme 2.2). This support ranged from assisting with short-term tasks to taking full responsibility for certain activities, such as managing the PO’s social media channels. Involving members in this direct way rather seemed to be done by smaller POs, with larger POs relying on their regional or national associations or external partners. External partners are needed to develop digital services that are specific to the PO (refer to the Subtheme 2.3: Public and Private Financial Assistance section and Table 3). Examples include the development of an app by which the members can communicate with each other, the design of a new PO website, and the provision of technical infrastructure, for instance, to establish patient registries. Furthermore, external partners are involved in developing health information for the POs’ digital formats. Health care professionals, mainly physicians, are asked to provide feedback as an advisory panel.

#### Subtheme 2.3: Public and Private Financial Assistance

According to some (6/46, 13%) of our interviewees, POs struggled to seek and maintain appropriate financial resources for planning and implementing various activities, such as digital services and projects. In their search for financial support, they applied for external funding from health care insurance and political actors (4/46, 9%). Furthermore, some (4/46, 9%) POs sought funding from pharmaceutical companies (subtheme 2.3 in Table 3).

### RQ 3: Involvement of PO Members in Digital Projects

#### Subtheme 3.1: Motivations to Establish and Sustain Involvement

In general, PO members repeatedly indicated a willingness to be involved in their POs’ digital initiatives (10/46, 22%; subtheme 3.1 in Table 3). They cited the benefits of the digital technologies being developed and the feeling that their involvement is making a difference as the main reasons for their involvement. PO representatives and members (13/46, 28%) stated that member involvement is necessary to consider users’ (ie, members’) needs and preferences regarding digital products. Despite the many positive perceptions and willingness vis-à-vis involvement, interviewees rarely referred to concrete examples of established participatory approaches, indicating a gap between aims or willingness and practice. For instance, only 3 (7%) out of 46 PO members mentioned participatory activities within their POs’ digital initiatives, such as members surveys or involving members in the design of a PO website. Regarding a PO’s size, interviewees from larger POs more often referred to the importance of and interest in involvement.

#### Subtheme 3.2: Types and Intensity of Involvement

PO members and representatives indicated rather unanimously that active involvement should take place early in the planning and implementation of the products. However, in contrast, they highlighted several times that currently, member involvement stays rather passive (13/46, 28%), for example, by participating in a member survey (4/46, 9%) or user testing (2/46, 4%). Some (2/46, 4%) representatives mentioned that they do offer members the opportunity to contribute more actively, for example, by serving on an advisory board for the development of a patient registry or on a research committee (subtheme 3.2 in Table 3). Unfortunately, members (7/46, 15%) indicated that they were not aware of such opportunities to become involved. This meshes with representatives, who said that members involved in such digitalization efforts tend to be those who already volunteer within their PO. Moreover, we found more examples of actual involvement practices from interviewees belonging to larger POs.

#### Subtheme 3.3: Requirements for Involvement

Because of this struggle to achieve “true,” that is, active and ongoing involvement, some (5/46, 11%) interviewees pointed out the need to improve the prerequisites and conditions for this. To achieve this, PO members repeatedly emphasized that digital projects should be set up in such ways that involvement is neither too time consuming nor too demanding, for example, in terms of specific professional or technical knowledge (13/46, 28%). This was considered crucial for the motivation of members to get involved (subtheme 3.3 in Table 3). The observation that members can bring specific expertise to digitalization projects, given their professional backgrounds, adds to the importance of involving them.

### RQ 4: Attitudes Toward Digitalization in POs

#### Overview

The impact of the digital activities undertaken by POs can be multiple. These impacts can occur at the individual level by enabling a broader group of members to participate in POs’ activities and services (eg, hybrid activities), at the organizational level by streamlining their operations (eg, digital membership system), at the research level by providing better integration into research (eg, digital patient registry), or at the political level by offering better representation in the health care system. In this section, we describe how our participants generally viewed the shift toward more digital communication and the increasing use of digital tools positively. Positive impacts due to digitalization seemed to be expressed more often by interviewees from larger POs, while negative aspects, that is, challenges and disadvantages, were equally expressed by interviewees from smaller and larger POs. However, certain aspects elicited mixed responses, including criticisms regarding how POs are digitally transforming their work.

#### Subtheme 4.1: Accelerating Management, Outreach, and Communication

Participants (14/46, 30%) mentioned several examples of successful digital initiatives. For instance, POs’ websites and, sometimes, social media channels have become key outreach tools to reach a wider audience and attract new members, especially younger people. Moreover, patient registries have been established to support research efforts, and during the COVID-19 pandemic, digital formats for face-to-face interaction and exchange were introduced, further enhancing communication. Furthermore, several (7/46, 15%) representatives highlighted how digital tools help to handle daily or regular tasks related to administration and management. Participants also mentioned the ability to network at a national or even European level through digital tools, for example by connecting with foreign sister organizations and research institutes that the PO had not previously worked with. Moreover, some (2/46, 4%) mentioned that costs can be reduced by moving from analog to digital formats.

#### Subtheme 4.2: Simplifying Access to PO Support Services

Many (30/46, 65%) participants described digital tools as helpful and convenient. PO representatives saw videoconferencing software as particularly valuable for simplifying communication or facilitating events. Representatives and members alike appreciated the opportunity to improve access for those who cannot attend PO meetings or events in person, for example, due to a physical impairment.

#### Subtheme 4.3: Supporting Health Research for People With Chronic and Rare Diseases

Patient registries operated by POs were seen as particularly beneficial and promising by several (11/46, 24%) participants. Registries were seen to advance health research and the development of new treatments. Valuing the potential benefits for themselves and others affected, several (10/46, 22%) PO members indicated that they would generally be willing to provide their data for use in such a patient registry.

#### Subtheme 4.4: Ineffective, Costly, and Time-Consuming Activities

Despite these various positive aspects, especially PO representatives (8/46, 17%) reported it as costly, requiring significant personnel and financial resources and expertise to implement the projects. This included, but was not limited to, large-scale projects, such as patient registries and mobile apps, for which these resources were not readily available. These challenges were seen as particularly significant for smaller, resource-limited POs. Some PO representatives referred to unsuccessful or failed digital projects (3/46, 7%), although failures were less frequently reported compared to successes (14/46, 30%). For example, a participant noted that the PO’s social media channels were not effective enough in reaching a wider audience. Another mentioned a chat forum that was discontinued after a while due to lack of use. The last example is the discontinuance of a patient registry at a smaller PO due to a lack of resources and expertise.

#### Subtheme 4.5: Challenging Personal Interaction and Access

Representatives and members alike stated that the value of in-person meetings cannot be fully captured by digital alternatives (14/46, 30%). Despite perceived advantages, digital technologies were mainly seen as supplements rather than replacements. In addition, several participants (18/46, 39%) stated that digital formats are less accessible to older PO members or those with medical conditions impairing cognition. Participants stated that these persons may lack access to important information. They lack the literacy to work with digital tools, have physical difficulties using digital equipment, or have concerns regarding the use of digital technologies in general.

#### Subtheme 4.6: Requiring Additional Efforts for Planning, Training, and Risk Management

Both representatives and members emphasized that POs need to make additional efforts to succeed in their digital transformation (7/46, 15%), which is particularly difficult to achieve for smaller POs (5/46, 11%). Several (15/46, 33%) participants stressed the importance of training members to use digital technologies effectively. Furthermore, they emphasized the importance of digital services meeting members’ preferences, providing clear benefits, and being user-friendly. Another key issue our participants brought up is data privacy, for example, of the data stored within patient registries or gathered by the use of digital tools, such as mobile apps. In this study, the participants considered comprehensive privacy policies important and necessary.

#### Subtheme 4.7: Encouraging Ambivalence or Fostering Undecidedness

Several (11/46, 24%) participants expressed difficulties evaluating the consequences of digital technologies. They were generally uncertain, weighing benefits against potential risks. This was exacerbated, as the digital tools had often only recently been implemented. Others acknowledged that while digital workflows and communication might be feasible and useful in POs, they involved a significant amount of work and financial resources, which were scarce for POs. Furthermore, some (7/46, 15%) representatives pointed out that while digital transformation within the organization was generally positive, this might not be the case for everyone involved in the process. Specifically, they perceived older PO members as being more reluctant to embrace digitalization in the PO, resulting in low motivation levels, skepticism, or even resistance. Moreover, some (5/46, 11%) indicated that the benefits would eventually outweigh the potential drawbacks.

## Discussion

### Principal Findings

This interdisciplinary interview study explored how German POs enact their digital transformation (RQ 1), who is involved in this process (RQ 2 and RQ 3), and how PO representatives and members evaluate past and current efforts (RQ 4). This study aimed to provide empirical evidence on the *ways of working* of one of the most important institutional actors in health care and research. While digital transformation is seen as promising and potentially transformative for the delivery of care and research, practical insights into this process, its requirements, and the challenges for POs are largely lacking.

First, POs are driving digitalization primarily in 4 key areas: communication, health education, health research, and administration. Communication efforts are the most widespread, with many POs having established *basic* digital formats, such as websites, social media channels, and videoconferencing. Digital health education also plays a central role as POs work to improve members’ digital health literacy and provide accessible information. Furthermore, some POs are engaged in health research through digital data collection and have begun to digitalize administrative tasks, although this is comparatively less common. Second, POs rely heavily on internal support and external collaboration to develop and sustain these digital activities. The participants in our study often referred to volunteer members and collaborations with external public and private partners to manage the necessary workload and resources. Third, while our interviewees considered the active involvement of members, for example, in the design of digital tools, desirable, it is not a *routine* in POs and depends much on the sufficient motivation of those involved. Fourth, the digital transformation in health care comes with a new task for POs: improving the digital health literacy of their members. Finally, PO representatives and members highlighted that digital transformation brings hope to reach a wider audience and attract younger people. However, its advantages may only be realized if several barriers can be overcome. These include, most obviously, a lack of financial resources, the need for digital training for staff and volunteers, and appropriately handling the reduction of face-to-face communication.

### Roles and Tasks of POs

Until now, there has been limited research on the roles and tasks of POs in general and especially in relation to digital transformation. Our findings contribute to the evidence base in several ways. For example, according to van de Bovenkamp et al [10], POs seek and depend on collaboration with external stakeholders for a variety of reasons, not least to secure material and immaterial resources, which are often limited, especially in smaller organizations [15]. However, our PO representatives and members were somewhat ambivalent about this fact. On the one hand, POs collaborate in the planning and implementation of digital activities relatively intensively, involving many volunteers (ie, PO members) and, less frequently, sister organizations, such as the national and regional associations. On the other hand, collaboration with external partners, such as pharmaceutical companies or health insurance companies, was often described as hampered. It was perceived that it does not serve *true* collaboration purposes, such as jointly developing a new digital service. Rather, collaboration is meant to secure needed resources or outsource work to external partners given their limited capabilities and skills. However, according to van de Bovenkamp et al [10], POs need to foster actual collaboration to promote their role in the health care system.

While Claus et al [11] state that POs could help to recruit patients for research studies, we found little evidence that POs are indeed using their digital resources for this purpose [12]. Our findings show that POs are using a wide variety of more basic digital tools, mainly for communication purposes. Those tools include websites, social media, and digital events resulting from the POs’ internal digitalization efforts. In comparison, the development and use of more elaborate digital tools, such as mobile apps or patient data registries, is less common. A lack of resources, both in terms of finances and skill set, is an important reason for this. Considering the relative novelty of the digital tools and the lack of experience and expertise within the POs to use these tools, a robust digital governance framework is necessary to further advance the digital transformation in POs [3]. Such a governance framework would serve the POs as a guideline in the development and maintenance of their digital activities and services to ensure compliance with their core aims and values.

Although we did not directly assess the impact of these digital activities, participants’ experiences and attitudes suggest that digital transformation has a range of effects. For instance, digital tools were perceived as essential for outreach and communication; helpful in facilitating daily administrative tasks; and, in some cases, even reducing costs. Some (11/46, 24%) participants also mentioned that tools, such as videoconferencing, provide better access to support services for members with physical disabilities. In contrast, digital activities were seen as requiring significant financial and human resources, and certain individuals, especially older individuals or those with lower digital literacy, were reported to face barriers in accessing digital tools, potentially leading to exclusion. Hence, the digital transformation in POs, as reported by participants, has a broad impact, including both positive and negative aspects.

### Involvement of PO Members

Our findings highlight a consensus among many PO members and representatives regarding the value of participatory approaches to enhance the usability of digital services. This is consistent with the reported general benefits of stakeholder engagement in the development of digital health tools, highlighting the critical role of this approach [26-28].

The interviews revealed a strong willingness among members to engage in digital initiatives, while relatively few (3/46, 7%) reported actual participatory approaches. Some studies, although not specific to the PO context, point to organizational capacity constraints, including time and financial resources, which hinder a more thorough use of participatory approaches [28,29]. Further examining such challenges in the context of POs can provide valuable insights into bridging the gap between intentions to get involved and actual involvement.

To tap into the full range of perspectives and skills of PO’s members, organizations need to design involvement methods, as our interview data point to the design of such approaches as a potential pivot point for increasing involvement. For example, participants emphasized the importance of clear benefits, manageable requirements, and early engagement as key factors in their decisions to engage. POs may consider these aspects when planning participatory approaches, as they have also been identified as key participatory design principles for stakeholder engagement in other digital health projects [30,31].

Furthermore, participatory approaches used in POs’ digital initiatives seem to focus predominantly on the initial stages of engagement, such as surveys and user testing. The prevalence of these methods in the PO landscape may be due to their relative organizational simplicity and lower demands on participants. More active, collaborative forms, such as advisory boards, were less frequently mentioned. This echoes a trend found in 2 reviews, which identified such collaborations in the participatory development of digital health tools but comparatively more frequently reported forms of involvement that tended toward more passive engagement [29,31]. Regarding the PO context, this may prompt further consideration of the applicability of active involvement or the potential reluctance of members to take on more control and responsibility.

Building on our findings, future research should explore the willingness of PO members to engage in their PO projects, their specific needs and expectations, and the organizational capacity to support and sustain active involvement. Such studies could guide the design and implementation of participatory approaches in POs’ digital initiatives or even establish frameworks to align member expectations with organizational realities.

### Ethical Aspects Related to Digital Transformation Within POs

Our findings highlight the ethical complexities that come with the digital transformation. POs are confronted with a wide range of ethical considerations regarding their digital projects and services as well as the processes of developing and maintaining them. Topics such as digital literacy, accessibility of digital tools, and data security [32-34] are highly relevant to the POs’ digital transformation processes.

Our findings show the importance of accessibility to the digital services POs provide for their members. Interestingly, digitalizing services can lead both to enhanced and diminished accessibility of the services in question. For an inclusionary approach, POs need to identify the respective groups that will and will not most likely benefit from the digital transformation. One group that was seen as potentially disadvantaged was older members because they might lose the connection to the activities of the PO. This may be due to a lack of resources, insufficient digital literacy, or preferences for analog formats. However, the use of digital tools may also increase the accessibility of PO services. For example, one group for which digital tools increase accessibility is persons unable to attend in-person meetings due to their ailment or disability. Therefore, there is ambivalence concerning accessibility, meaning that POs have to carefully weigh their options and try to forge a path that includes all their members.

Furthermore, a just allocation of scarce resources and collaboration with external partners were considered important. Previous research has found that POs rely heavily on third-party funding; however, there is a lack of transparent information about potential conflicts of interest [18]. POs are challenged with the task of securing enough resources to allow them to develop and maintain their digital activities while remaining independent of undue influences. Their political and economic independence is especially important to maintain integrity and trustworthiness. In this regard, careful consideration is needed when choosing an external partner to safeguard the PO’s independence. However, such considerations might be hampered by a lack of choices.

Persons in POs contributing to digitalization projects may easily find themselves in attitudinal and judgmental conflicts when weighing the positive and negative sides of those projects. This stresses the need for an ethical governance framework for POs’ digital transformation.

### Strengths and Limitations

We were able to include participants from 19 individual POs, which represent a range of target groups, address various aspects in the field of health care and medical issues, and are based in different regions of Germany. By including POs from distinct places, we aimed to reduce potential bias from a concentration of organizations in certain regions and provide a more balanced view regarding digitalization. We believe that the diversity of the included POs may have contributed to a broader range of perspectives on POs’ digital transformation while allowing us to clearly identify the most prominent practices, actors, and attitudes. However, as digitalization activities vary considerably between POs, especially regarding their digital educational services, the development of their own technologies, and research activities, there could be a greater differentiation of these digitalization activities and the actors involved depending on the size of a PO, the topics dealt with, and the level of digitalization within each organization. In addition, the observed variations in digital activities are based on subjective reports and may not capture all initiatives. Furthermore, because we included only POs that demonstrated some level of digital activity, the perspectives of those still at the very beginning of their digital transformation or those that have chosen not to engage in this process are not represented in our study; therefore, it may be assumed that for such POs, the extent of digitalization is lower. Given our limited personnel resources, we did not return interview transcripts and the eventual findings to the participants for comments. However, additional steps to increase the validity of the findings were performed, such as peer checks of the coded text segments and peer interpretation of the findings (refer to the Methods section for more validating steps). Our study can be a starting point for further such analyses.

### Conclusion

Our study provides insights into the state of digital transformation in German POs. We found that digitalization efforts are particularly evident in 4 core areas: communication, administration, health education, and health research. These processes involve multiple professional and nonprofessional actors, including permanent staff, volunteers, and external partners. Member involvement varies in scope and form, with a high level of willingness to engage. While representatives and members generally view digitalization positively, they also identify barriers, such as resource limitations and accessibility challenges, for certain groups.

In conclusion, German POs are currently investing considerable resources to engage in digital transformation processes, ranging from smaller projects to more sophisticated initiatives, and involving a wide range of stakeholders and individuals. These efforts and collaborations highlight the need for POs to establish more comprehensive ethical governance frameworks to clarify the goals of digitalization, determine what is needed to achieve them, and define how to engage with different stakeholders, including setting clear roles and motivations for their members to become involved. Public institutions could assist POs with these multiple tasks and requirements, for example, by providing training, mentorship, and facilitating networking and sharing among POs.

## Appendix

Multimedia Appendix 1 COREQ (Consolidated Criteria for Reporting Qualitative Research) checklist.

Multimedia Appendix 2 Overview of interview and focus group questions.

## Abbreviations

COREQ: Consolidated Criteria for Reporting Qualitative Research
PO: patient organization
RQ: research question

## References

[1] Stoumpos AI, Kitsios F, Talias MA (2023). Digital transformation in healthcare: technology acceptance and its applications. Int J Environ Res Public Health.

[2] Kraus S, Schiavone F, Pluzhnikova A, Invernizzi AC (2021). Digital transformation in healthcare: analyzing the current state-of-research. J Bus Res.

[3] (2020). Digital transformation: shaping the future of European healthcare. Deloitte Centre for Health Solutions.

[4] Konttila J, Siira H, Kyngäs H, Lahtinen M, Elo S, Kääriäinen M, Kaakinen P, Oikarinen A, Yamakawa M, Fukui S, Utsumi M, Higami Y, Higuchi A, Mikkonen K (2019). Healthcare professionals' competence in digitalisation: a systematic review. J Clin Nurs.

[5] Burmann A, Tischler M, Faßbach M, Schneitler S, Meister S (2021). The role of physicians in digitalizing health care provision: web-based survey study. JMIR Med Inform.

[6] Brown EE (2021). Assessing the quality and reliability of COVID-19 information on patient organization websites. Front Commun.

[7] Pauer F, Litzkendorf S, Göbel J, Storf H, Zeidler J, Graf von der Schulenburg JM (2017). Rare diseases on the internet: an assessment of the quality of online information. J Med Internet Res.

[8] van de Bovenkamp HM, Trappenburg MJ, Grit KJ (2010). Patient participation in collective healthcare decision making: the Dutch model. Health Expect.

[9] Souliotis K, Agapidaki E, Peppou LE, Tzavara C, Varvaras D, Buonomo OC, Debiais D, Hasurdjiev S, Sarkozy F (2018). Assessing patient organization participation in health policy: a comparative study in France and Italy. Int J Health Policy Manag.

[10] van de Bovenkamp H, de Graaff B, Kalthoff K, Bal R (2024). The patient representation struggle during the COVID-19 pandemic: missed opportunities for resilient healthcare systems. Health Expect.

[11] Claus EB, Feliciano J, Benz LS, Calvocoressi L (2020). Social media partnerships with patient organizations for neuro-oncology patient recruitment. Neurooncol Pract.

[12] Gentilini A, Miraldo M (2023). The role of patient organisations in research and development: evidence from rare diseases. Soc Sci Med.

[13] Mitchell D, Geissler J, Parry-Jones A, Keulen H, Schmitt DC, Vavassori R, Matharoo-Ball B (2015). Biobanking from the patient perspective. Res Involv Engagem.

[14] Rabeharisoa V (2003). The struggle against neuromuscular diseases in France and the emergence of the "partnership model" of patient organisation. Soc Sci Med.

[15] Sienkiewicz D, van Lingen C (2017). The added value of patient organisations. European Patients Forum.

[16] Madanian S, Nakarada-Kordic I, Reay S, Chetty T (2023). Patients' perspectives on digital health tools. PEC Innov.

[17] Nguyen CQ, Kariyawasam D, Alba-Concepcion K, Grattan S, Hetherington K, Wakefield CE, Woolfenden S, Dale RC, Palmer EE, Farrar MA (2022). 'Advocacy groups are the connectors': experiences and contributions of rare disease patient organization leaders in advanced neurotherapeutics. Health Expect.

[18] Mikami K, Sturdy S (2017). Patient organization involvement and the challenge of securing access to treatments for rare diseases: report of a policy engagement workshop. Res Involv Engagem.

[19] Patient-centered digitalization: an ethical analysis of the role of patient organizations as actors in the context of digitalization in health-related research and care (PANDORA). Pandora Research Consortium.

[20] Patient-centered digitalization: an ethical analysis of the role of patient organizations as actors in the context of digitalization in health-related research and care (PANDORA). PANDORA forscht.

[21] Tong A, Sainsbury P, Craig J (2007). Consolidated criteria for reporting qualitative research (COREQ): a 32-item checklist for interviews and focus groups. Int J Qual Health Care.

[22] Rach C, Lukas J, Müller R, Sendler M, Simon P, Salloch S (2020). Involving patient groups in drug research: a systematic review of reasons. Patient Prefer Adherence.

[23] Patterson AM, O'Boyle M, VanNoy GE, Dies KA (2023). Emerging roles and opportunities for rare disease patient advocacy groups. Ther Adv Rare Dis.

[24] Rauter CM, Wöhlke S, Schicktanz S (2021). My data, my choice? - German patient organizations' attitudes towards big data-driven approaches in personalized medicine. An empirical-ethical study. J Med Syst.

[25] Kuckartz U (2016). Qualitative Inhaltsanalyse. Methoden, Praxis, Computerunterstützung.

[26] Jahnel T, Schüz B (2020). [Participatory development of digital public health: tension between user perspectives and evidence]. Bundesgesundheitsblatt Gesundheitsforschung Gesundheitsschutz.

[27] Hochmuth A, Exner AK, Dockweiler C (2020). [Implementation and participatory design of digital health interventions]. Bundesgesundheitsblatt Gesundheitsforschung Gesundheitsschutz.

[28] Clemensen J, Rothmann MJ, Smith AC, Caffery LJ, Danbjorg DB (2017). Participatory design methods in telemedicine research. J Telemed Telecare.

[29] Moore G, Wilding H, Gray K, Castle D (2019). Participatory methods to engage health service users in the development of electronic health resources: systematic review. J Particip Med.

[30] Voorheis P, Petch J, Pham Q, Kuluski K (2023). Maximizing the value of patient and public involvement in the digital health co-design process: a qualitative descriptive study with design leaders and patient-public partners. PLOS Digit Health.

[31] Sanz MF, Acha BV, García MF (2021). Co-design for people-centred care digital solutions: a literature review. Int J Integr Care.

[32] Vayena E, Haeusermann T, Adjekum A, Blasimme A (2018). Digital health: meeting the ethical and policy challenges. Swiss Med Wkly.

[33] Maeckelberghe E, Zdunek K, Marceglia S, Farsides B, Rigby M (2023). The ethical challenges of personalized digital health. Front Med (Lausanne).

[34] Brall C, Schröder-Bäck P, Maeckelberghe E (2019). Ethical aspects of digital health from a justice point of view. Eur J Public Health.

