# Supplementary material for: Digital Transformation in Patient Organizations: Interview and Focus Group Study
Source: J Med Internet Res. 2025 Feb 13;27:e62750. doi: 10.2196/62750 (PMC11888101; doi:10.2196/62750)
Supplement: Multimedia Appendix 1 [file jmir_v27i1e62750_app1.pdf]

COREQ

Consolidated criteria for reporting qualitative studies (COREQ): 32-item checklist
Developed from: Tong A, Sainsbury P, Craig J. Consolidated criteria for reporting qualitative research (COREQ): a 32-item checklist for interviews and focus groups. *International Journal for Quality in Health Care*. 2007. Volume 19, Number 6: pp. 349 – 357

No. Item	Guide questions/description	Reported on page #
**Domain 1 Research team and reflexivity**		
Personal Characteristics		
1. Interviewer/ facilitator	Which author/s conducted the interview or focus group?	# 4
2. Credentials	What were the researcher's credentials? E.g. PhD, MD	Title page
3. Occupation	What was their occupation at the time of the study?	# 4
4. Gender	Was the researcher male or female?	# 4
5. Experience and training	What experience or training did the researcher have?	# 4
**Relationship with participants**		
6. Relationship established	Was a relationship established prior to study commencement?	# 5
7. Participant knowledge of the interviewer	What did the participants know about the researcher? e.g. personal goals, reasons for doing the research	# 6
8. Interviewer characteristics	What characteristics were reported about the interviewer/facilitator? e.g. Bias, assumptions, reasons, and interests in the research topic	# 5
**Domain 2 Study design**		
**Theoretical framework**		
9. Methodological orientation and Theory	What methodological orientation was stated to underpin the study? e.g. grounded theory, discourse analysis, ethnography, phenomenology, content analysis	# 4, 5
**Participant selection**		
10. Sampling	How were participants selected? e.g. purposive, convenience, consecutive, snowball	# 4
11. Method of approach	How were participants approached? e.g. face-to-face, telephone, mail, email	# 4
12. Sample size	How many participants were in the study?	# 6
13. Non-participation	How many people refused to participate or dropped out? Reasons?	n.a.
**Setting**		
14. Setting of data collection	Where was the data collected? e.g. home, clinic, workplace	# 4
15. Presence of non-participants	Was anyone else present besides the participants and researchers?	# 5

16. Description of sample	What are the important characteristics of the sample?	Table 1
**Data collection**		
17. Interview guide	Were questions, prompts, guides provided by the authors? Was it pilot tested?	# 4-5
18. Repeat interviews	Were repeat interviews carried out? If yes, how many?	# 5
19. Audio/visual recording	Did the research use audio or visual recording to collect the data?	# 5
20. Field notes	Were field notes made during and/or after the interview or focus group?	# 5
21. Duration	What was the duration of the interviews or focus group?	Table 1
22. Data saturation	Was data saturation discussed?	# 5
23. Transcripts returned	Were transcripts returned to participants for comment and/or correction?	# 17
**Domain 3 Analysis and findings**		
**Data analysis**		
24. Number of data coders	How many data coders coded the data?	# 5
25. Description of the coding tree	Did authors provide a description of the coding tree?	Table 2
26. Derivation of themes	Were themes identified in advance or derived from the data?	# 5
27. Software	What software, if applicable, was used to manage the data?	# 5
28. Participant checking	Did participants provide feedback on the findings?	# 17
**Reporting**		
29. Quotations presented	Were participant quotations presented to illustrate the themes/findings? Was each quotation identified? e.g. participant number	Table 2
30. Consistency	Was there consistency between the data and the findings?	n.a.
31. Major themes	Were major themes clearly presented in the findings?	# 7-13
32. Minor themes	Is there a description of diverse cases/minor themes?	# 7-13
