# Supplementary material for: Digital Transformation in Patient Organizations: Interview and Focus Group Study
Source: J Med Internet Res. 2025 Feb 13;27:e62750. doi: 10.2196/62750 (PMC11888101; doi:10.2196/62750)
Supplement: Multimedia Appendix 2 [file jmir_v27i1e62750_app2.pdf]

Electronic supplement 2 – overview of questions

The following questions have originally been asked in German.

Project Hamburg

General aspects about digitalization

- What does "digitalisation" in medical research and care mean to you? What specific examples do you know or have you dealt with in the past?
- What experience do you or your organisation have with digitalisation in the field of medical research and care?
- What do you understand by health literacy? And what level of expertise do you consider necessary for you or other people to be able to participate in digital health projects and technologies?
- What does digital health literacy mean to you in this context?

Digital products, projects, processes

- Have any specific complaints or problems been raised by your members in previous digitisation projects in which your organisation has been involved? If so, could you explain them?
- Have you involved members of your organisation in digitalisation processes or designed them together?
- What measures have been taken to increase members' confidence in digitalisation projects?
- Patients are increasingly using health-related digital apps themselves or participating in e-health and digitalisation initiatives such as digital patient registers, telehealth and monitoring via apps. What changes do you think this means for the role of patients?
- In which areas could patients be more actively involved in research/digitalisation projects? Where do you see the greatest challenges in this respect?
- In the context of research initiatives that analyse large amounts of data, there are efforts to replace the individual informed consent of participants with broad consent. What is your organisation's position on such a proposal?
- Are there any other experiences and opinions that you would like to share that have not been addressed so far?

Ethical aspects

- What opportunities and risks does your organisation expect from the use of digital technologies in the medical field?
- What ethical issues are important to you in the use of digital technologies in the medical and/or research sector?
- In your organisation's opinion, is the current regulation for the use of digital technologies and the use of personal data in the medical field sufficient to protect those affected? What could or should be improved?
- How would PO change as a result of digitalisation? Here we are interested in both the services offered and the procedures and processes within an organisation.
- In your opinion, what responsibilities do participating organisations have when implementing digital health projects?

Project Hannover

General aspects about digitalisation

- What do you associate with the term digitalisation?
- How would you describe digitalisation, i.e. the use of digital media and applications, in your organisation?
- How important or helpful do you find the use of digital technologies in your organisation?
- Have you noticed that the use of these technologies has changed processes, structures or services in your organisation?
- What could and should develop in a new or different way in the future?
- One digital application that the patient organisation has developed or was involved in developing is (...). As I will now refer to this app in my questions, I would first like to know whether you know (...) and whether you currently use this app yourself? If so, why and what for?

Chances, challenges, ethical aspects

- When you think about the digitalisation processes/projects in the patient organisation described above and in the future, what advantages and disadvantages do you personally see? Are there any aspects that you have concerns about? What do you find particularly good?
- When it comes to the advantages and disadvantages of digitalisation, questions often arise, for example about self-determination, responsibility, trust, etc. Can you name other such or similar topics or questions when you think about the use of (...)?
- Would you say that your organisation takes these (ethical) aspects into account or addresses them when it comes to (...) or other digitalisation processes? If so, how?

Participation and collaboration

- When the app XY was developed and/or introduced in your organisation, did your organisation ask you for support or were you involved in this process? If yes, to what extent? If not, would you consider this important?
- Is it important to you which stakeholders are involved in the development or provision of a digital application when you are thinking about using it?

Digital literacy

- How do you feel about using the XY app (e.g. safe/unsafe)?
- Do you have special needs or do you see certain hurdles for yourself in order to be able to make good use of (...) or other digital offerings from your organisation?

Digitalisation in health care

- When you think about digitalisation in the healthcare sector, what are the advantages and disadvantages for you?
- What would be particularly important to you personally in order to make the best possible use of the advantages of digitalisation in the healthcare sector?

Project Göttingen

General aspects of digitalisation

- App development is time-consuming, expensive and ties up human resources: why should a patient organisation do this?
- What is your motivation for taking on such a project?

Challenges

- If you now imagine that you are developing an e-health product with your PO, for example an app, or if you think back to what it was like when you were doing this: Do you think you would encounter any challenges?
- If you take another look at the challenges that have just been discussed here: Who (inside your PO and outside) would you say particularly face these challenges?

Chances

- If you imagine that you and your PO are considering developing an e-health product, or if you think back to how it was, what could motivate you to take this step? Do you have concrete hopes for it? Can the e-health product achieve something that would otherwise be impossible?
- If you take another look at the opportunities that have just been discussed here: Who would you say these opportunities are particularly for (inside your PO and outside)?
- Here, too, I would like to sort things out with you and think about the different areas in which there are opportunities. We have not thought of any categories in advance, but would like to ask you to sort them.

Comparison of both

- If we now take a look at our list, which of the opportunities and challenges we have talked about would you rate as particularly relevant and which as less relevant?

Ethical aspects

- To what extent (and why?) do you find the following points relevant when it comes to digital products from POs?
    - Self-determination
    - Responsibility
    - Solidarity
    - New forms of solidarity (data donation)
    - Endangering the community of solidarity
    - Conflicts of interest
    - Equity in access
    - Fairness in distribution
- I would like to talk to you about how to deal with these challenges. Or how you dealt with them when they arose?

Support

- What could a research project like Pandora do to support PO with digitalisation?
